# Probiotics, Nuclear Receptor Signaling, and Anti-Inflammatory Pathways

**DOI:** 10.1155/2011/971938

**Published:** 2011-07-26

**Authors:** Sonia S. Yoon, Jun Sun

**Affiliations:** ^1^Division of Gastroenterology and Hepatology, Department of Medicine, Strong Memorial Hospital, University of Rochester Mediacal Center, University of Rochester, Rochester, NY 14642, USA; ^2^Department of Microbiology and Immunology, University of Rochester, P.O. Box 646, 601 Elmwood Avenue, Rochester, NY 14642, USA

## Abstract

There is increased investigation of the human microbiome as it relates to health and disease. Dysbiosis is implicated in various clinical conditions including inflammatory bowel disease (IBD). Probiotics have been explored as a potential treatment for IBD and other diseases. The mechanism of action for probiotics has yet to be fully elucidated. This paper discusses novel mechanisms of action for probiotics involving anti-inflammatory signaling pathways. We highlight recent progress in probiotics and nuclear receptor signaling, such as peroxisome-proliferator-activated receptor gamma (PPAR*γ*) and vitamin D receptor (VDR). We also discuss future areas of investigation.

## 1. Introduction

Probiotics are ingestible microorganisms with health benefits. Increased interest in the intestinal microbiome and its effect on health and disease is evidenced by the concomitant increase in peer-reviewed clinical trials investigating probiotics as therapy since 1999 [1]. Studies of the various signaling pathways involved in the response to bacteria and inflammation have led to a more detailed understanding of mechanisms and actions of probiotics. This paper discusses progress in understanding how probiotics contribute to intestinal mucosal function, particularly in relation to anti-inflammatory signaling pathways.

## 2. Intestinal Microflora

The intestinal microflora, as a whole, serves important functions in metabolism, intestinal epithelial cell function and health, immunity, and inflammatory signaling [2, 3]. Recently, there has been increasing interest in the role of the intestinal microflora and its total genetic composition, together referred to as the microbiome in the development, maintenance, and perpetuation of various clinical conditions, both intestinal and extraintestinal.

Dysbiosis has been implicated in various clinical conditions including atopy, irritable bowel syndrome (IBS), colorectal cancer, alcoholic liver disease in animal and human studies, obesity and other metabolic disorders, and chronic inflammatory diseases such as IBD [4–11]. Decreased diversity of the intestinal microbiota was seen in fecal samples obtained from children who subsequently developed allergic disease [6, 7]. Altered microbiota composition in colon cancer patients when compared to patients with normal colonoscopies and in patients with IBS compared to unaffected patients has also been demonstrated [5, 9]. Alcohol feeding resulted in enteric bacterial overgrowth in a mouse model [8]. The role of the microbiota in obesity has been extensively studied and carefully reviewed in the literature [12, 13]. Microbial composition in IBD patients with ulcerative colitis (UC) or Crohn's disease (CD) as compared to unaffected individuals has been studied and shows decreased diversity [4, 14–19]. This altered microflora may have significant implications for the intestinal milieu, with as yet incompletely understood effects. The pathogenesis of IBD likely involves a combination of factors including intestinal dysbiosis in conjunction with environmental factors in a genetically susceptible host [20]. 

Based on the concept of a dysregulated or dysfunctional microbiota in disease, various methods to attenuate the effects of an altered microbiome have been attempted.

## 3. Probiotics

“Probiotics” were first described in the literature by Lilly and Stillwell in 1965 as growth-promoting factors produced by certain microorganisms [21] although it may have been described as early as 1908 [22]. Recently, probiotics were defined as “live organisms which, when consumed in adequate amounts as part of food, confer a health benefit on the host” (Report of a Joint FAO/WHO Expert Consultation on Evaluation of Health and Nutritional Properties of Probiotics in Food Including Powder Milk with Live Lactic Acid Bacteria (October 2001), “Health and Nutritional Properties of Probiotics in Food Including Powder Milk with Live Lactic Acid Bacteria”, Food and Agriculture Organization of the United Nations, World Health Organization). The mechanisms of action of probiotics include immune modulation, direct effect on commensal and pathogenic bacteria to inhibit infection and restore homeostasis, and modification of pathogenic toxins and host products [23]. The efficacy of probiotics in various clinical conditions both in the pediatric and adult patient population has been extensively studied and carefully reviewed [1, 19, 24–32]. 

 Rectal infusion of normal stool via enemas to treat pseudomembranous colitis has been described as early as 1958 [70]. Infusion of stool via nasogastric tube to the small intestine or via colonoscopy to the colon for CDAD has also been described and shows high response rates [71–74]. A recent study showed that fecal bacteriotherapy was effective in relief of clinical symptoms in a patient with recurrent CDAD and that this was accompanied by the repopulation of the diseased intestinal microbiota with beneficial species that were diminished pretreatment [75]. Other methods to supply live, nonpathogenic organisms to the intestinal microbiota in AAD and CDAD include orally administered probiotics. The efficacy of various probiotic formulations in AAD and CDAD has been extensively studied and carefully reviewed [1]. A recent study showed that the probiotics *Lactobacillus acidophilus* and *Lactobacillus casei* were well tolerated and effective in reducing the risk of the development of AAD and CDAD [76]. The utility of the probiotic yeast *Saccharomyces boulardii* for a variety of conditions including traveler's diarrhea, enteral nutrition-associated diarrhea, AAD, and CDAD has been investigated, and according to a recent meta-analysis, strong evidence exists for advocating its use in traveler's diarrhea and AAD [77]. Recent trials using *Bifidobacterium bifidum* and *Saccharomyces boulardii* demonstrated improvement in clinical IBS symptoms and quality of life [78, 79], and several reviews of the evidence for the utility of probiotics in IBS have been published [80–82].

For IBD therapy, treatment with different strains of probiotics has shown varied results. Small trials have shown promise for probiotic use in the induction and maintenance of remission in UC. VSL#3 has been shown to be safe and effective in the treatment of acute mild to moderately active UC [83]. Patients with mild to moderate UC unresponsive to conventional therapy achieved a combined induction remission/response rate of 77% with treatment with VSL#3 [84]. *E. coli *Nissle 1917 was found to be effective and equivalent to mesalazine in maintaining remission in UC [85]. In another study, *Lactobacillus rhamnosus* GG (LGG) was equivalent to mesalazine in the maintenance of remission in UC, however, appeared to be more effective in prolonging the relapse-free time [86]. Evidence also exists for the role of probiotics in prophylaxis of pouchitis after surgery in UC patients as well as induction of remission in chronic pouchitis [87, 88].

Studies of probiotic use in induction and maintenance of remission and prevention of postoperative recurrence in CD have been less consistent than those for UC. A small study of LGG for the prevention of recurrence after surgery in CD did not show any improvement over placebo [89]; however, Sa*ccharomyces boulardii* appears useful in maintaining remission in CD [90, 91]. The progress in the use of probiotics for IBD has been carefully reviewed [92, 93]; however, there remains a relative lack of well-designed, large, randomized, placebo-controlled trials. 

Several barriers exist to advocating broad use of probiotics in clinical practice, not least of which is the considerable heterogeneity in the experimental designs with respect to species and strains of probiotics and the various animal models utilized [94]. Although clinical trials examining the role of probiotics in the treatment and/or prevention of AAD, CDAD, IBD including UC, CD, and pouchitis, necrotizing enterocolitis, infectious gastroenteritis, radiation-induced enteritis, and colitis, IBS and various atopic diseases have been reported [1, 24, 25, 28, 29, 31, 87, 95–97]; in many cases, results have been inconsistent, and large, well-designed trials are lacking. An additional complicating factor pertains to issues of quality control. Determining whether a commercially available probiotic actually contains the live organisms it purports to contain and determining if there is rational selection of component probiotic strains in “cocktails” are issues that must be considered [22]. Future research to refine techniques to accurately identify “normal” and “diseased” microbiota and to further elucidate the specific effects and mechanisms of actions of individual probiotic strains will aid in optimizing therapeutic efficacy. 

## 4. Mechanisms for Probiotics in Anti-Inflammation

There has been and continues to be considerable research in delineating the underlying mechanisms by which probiotics exert their beneficial effects. The mechanisms regulating the function of probiotics are very diverse. It is well accepted that probiotics use distinct cellular and molecular mechanisms, including blocking pathogenic bacterial effects, regulating immune responses, and altering intestinal epithelial homeostasis by promoting cell survival, enhancing barrier function, and stimulating protective responses [32].

Table 1 outlines representative publications on probiotic mechanisms of actions. The probiotic-host interaction is complex and further complicated by the fact that certain probiotic effects appear to be species and strain specific. Different probiotics have been shown to exert both pro-inflammatory [98] and anti-inflammatory effects on dendritic cells [99]. A recent study demonstrated that the anti-inflammatory effect of certain *lactobacilli* is via NOD2-mediated signaling [100]. NOD2/CARD15 is a member of a superfamily of genes involved in intracellular bacterial recognition and has been identified as an important susceptibility gene for CD [101, 102]. The authors speculate that the inconsistent clinical results of *lactobacilli* use in patients with CD may be related to a relative deficiency of NOD2. Probiotic effect on the innate immune responsive pathways including toll-like receptor (TLR), nuclear factor kappa B (NF-*κ*B), mitogen-activated protein kinase (MAPK), c-Jun NH2-terminal kinase (JNK) has been extensively investigated (Table 1). Activation of specific TLRs also appears to be species specific [47, 48]. The action of *E. coli* Nissle 1917 on Caco-2 cells was found to be mediated by flagellin possibly via a TLR pathway [103]. The probiotic-induced effect on the NF-*κ*B signaling pathway is well represented in the literature and is generally characterized by inhibition (Table 1). 

Defective epithelial barrier function has been implicated in IBD and can predict relapse during clinical remission [104–109]. One way by which probiotics have been shown exert their action is by stabilizing tight junctions (TJs) and enhancing barrier function of intestinal epithelial cells (Table 1). 

Abnormal STAT/suppressor of cytokine signaling (SOCS) signaling has been demonstrated in CD patients [110], and probiotics are also shown to modulate the JAK-STAT signaling in human placental trophoblast cells [111]. Increasing evidence further demonstrates that metabolism, xenobiotics, and nuclear receptor signaling are involved in the action of probiotics [67, 68]. 

Induction of heat shock proteins (HSPs) and endogenous antimicrobial peptides (defensins) via activation of NF-*κ*B, MAPK, and JNK has also been linked to probiotic action [35, 41, 43]. Since defensins are implicated in the pathogenesis of IBD, increased expression by probiotics provides a possible mechanism for clinical efficacy seen in certain IBD patients and deserves further study.

## 5. Defensins and Nuclear Receptor Signaling

Defensins are a class of endogenous antimicrobial peptides involved in innate immunity which is highly evolutionarily conserved and represents a primary line of defense against various microbial pathogens [112–114]. Antimicrobial peptides are widely distributed throughout the animal and plant kingdom, and despite their evolutionary heritage, remain effective antimicrobial agents [114]. This is due, in large part, to their mechanism of action involving membrane disruption and pore formation, which is not easily exploited by pathogens to confer resistance [112–115]. Important antimicrobial peptides in humans include defensins, cathelicidins, lysozymes, and other antimicrobial antiproteases [116]. There are three known defensin subfamilies; *α* and *β* defensins are expressed mainly in immune cells and epithelial cells while the *θ* defensin is found mainly in immune cells of the Rhesus macaque [117, 118]. In the gastrointestinal tract, *β* defensin expression is seen in multiple sites, whereas *α* defensin expression is largely in the small intestine [119]. In the uninflamed colon, human *β* defensin 1 is the predominant defensin and human *β* defensin 2 and 3 are induced with inflammation or infection [120]. In mice lacking functional cryptidins (murine *α* defensins), increased survival and virulence of orally administered bacteria were seen and intestinal peptide preparations had decreased antimicrobial activity [121].

The possible role of a deficiency in defensins in the pathogenesis of IBD has been proposed [116, 122]. The Paneth cells of the small intestine are the major source of endogenous antimicrobials, including *α* defensins [102]. In addition, The Paneth cells have been shown to express NOD2 [123]. In patients with ileal CD, human *α* defensin 5 and 6 production is reduced, and this effect is magnified in those patients with a concomitant NOD2 mutation [124]. For *β* defensins, CD patients with colonic disease exhibit normal levels of *β* defensin 2 and 3 whereas UC patients have increased levels, suggesting a role of failure of *β* defensin induction in the pathogenesis of CD [125]. Constitutive human *β* defensin 1 expression is reduced in CD patients with colonic involvement independent of inflammation, and recently, the maintenance of constitutive *β* defensin expression was shown to be activated by the nuclear receptor peroxisome-proliferator-activated receptor gamma (PPAR*γ*) [122].

Further contributing to the effect of a defensin deficiency in the pathogenesis of IBD may be the diminished diversity of the intestinal microbiota seen in IBD patients. The interaction of commensal bacteria with antimicrobial peptide synthesis is not well understood; however, it has been suggested that commensal bacteria provide chronic stimulation of epithelial cells to produce antimicrobial peptides at levels sufficient to kill microbial pathogens [114, 126]. 

Probiotics, but not fecal isolates, have been shown to induce human *β* defensin 2 in intestinal epithelial cells [41, 42]. Wehkamp et al. and Schlee et al. have reported that NF-*κ*B and activator protein-1 (AP-1) mediate induction of human *β* defensin 2 in intestinal epithelial cells by the probiotic *E. coli* Nissle 1917 and VSL#3 [41, 42].

Interestingly, nuclear receptors are known to regulate the expressions of defensins [122, 127]. Nuclear receptors represent a class of intracellular transcription factors activated by ligands which can directly interact with DNA; as a result, nuclear receptors play significant roles in the regulation of metabolic, reproductive, developmental, and immune processes [128–131]. Nuclear receptors regulate transcriptional activity by several distinct mechanisms, including “ligand-dependent transactivation, ligand-independent repression, and ligand-dependent transrepression” although the range of transcriptional activities of each nuclear receptor varies and even the transcriptional effects of a single nuclear receptor may be cell specific [132]. A detailed discussion of nuclear receptors and their mechanisms of action is beyond the scope of this article; however, further discussion of two nuclear receptors (peroxisome-proliferator-activated receptor gamma (PPAR*γ*) and vitamin D receptor (VDR)) with putative roles in inflammation is warranted. 

PPAR*γ* is a member of a class of nuclear receptors that form obligate heterodimers with the retinoid X receptor (RXR) [129]. The PPAR family has been shown to affect various cellular functions including “adipocyte differentiation, fatty-acid oxidation, and glucose metabolism” [129]. PPAR*γ* is highly expressed in the large intestine [133], and its activation has been shown to be protective in animal models of colitis [134, 135]. Decreased PPAR*γ* expression in UC patients has been shown [136], and the anti-inflammatory compound 5-aminosalicylic acid (5-ASA) commonly utilized in IBD therapy was shown to be a PPAR*γ* agonist, thereby establishing a possible mechanism by which it exerts its anti-inflammatory effects [137]. PPAR*γ* also plays a role in the maintenance of “constitutive epithelial expression of a subset of *β* defensins in the colon” [122].

## 6. Vitamin D and Vitamin D Receptor (VDR)

Vitamin D receptor (VDR) is a nuclear receptor that mediates most known functions of 1,25-dihydroxyvitamin D (1,25(OH)_2_D_3_), the active form of vitamin D [138]. VDR heterodimerizes with RXR once VDR is activated by 1,25(OH)_2_D_3_. VDR binds to the vitamin D response element in the target gene promoter to regulate gene transcription [139]. VDR downstream target genes include antimicrobial peptides such as cathelicidin and *β* defensin. 

VDR is critical in regulating intestinal homeostasis by preventing pathogenic bacterial invasion, inhibiting inflammation, and maintaining cell integrity [140–145]. Vitamin D directly modulates the T-cell receptor (TCR) [146], and vitamin D has also been shown to downregulate the expression of proinflammatory cytokines and have regulatory effects on autophagy and various immune cells including T cells, B cells, macrophages, dendritic cells, and epithelial cells [147, 148]. It has been reported that 1,25(OH)_2_D_3_ suppresses the development of IBD in animal models [149]. Deficiency of 1,25(OH)_2_D_3_ has been reported in patients with IBD [150, 151], and, recently, using a novel vitamin D bioavailability test, vitamin D deficiency or insufficiency was seen in more than 70% of patients with quiescent CD [152]. Given the diverse immune functions of vitamin D, deficient levels may have important implications for the development and maintenance of intestinal homeostasis. A possible role of vitamin D status and VDR signaling in modulating the effects of intestinal microflora in other conditions such as asthma and obesity has been suggested [100]. While present literature has primarily focused on elucidating the immunoregulatory effects of vitamin D, there is a paucity of data on the status and function of VDR [147]. In addition, probiotic-induced modulation of anti-inflammatory VDR signaling in colitis remains virtually unexplored.

Recent studies indicate that VDR−/− mice have increased bacterial loading in the intestine [145, 153]. Our microarray data found that VDR signaling responds to pathogenic *Salmonella* in intestinal colitis *in vivo *[154]. Data from a recent study demonstrate that bacterial stimulation, both commensal and pathogenic, regulates VDR expression and location and that VDR negatively regulates bacterial-induced intestinal NF-*κ*B activation [153]. In general, probiotic-induced nuclear receptor signaling is not well characterized. The probiotic VSL3# was associated with nuclear receptor signaling in the IL10−/− colitis model [67]. Nuclear receptors have been shown to negatively regulate bacterial-stimulated NF-*κ*B activity in intestinal epithelium [153, 155]. Our recent data show probiotic treatment is able to enhance VDR expression and activity in the host. An increase in VDR expression and a concomitant increase in cathelicidin mRNA in cultured intestinal epithelial cells when treated with *Lactobacillus plantarum* were seen [156]. We used a probiotic monoassociated pig model to assess the probiotic effect on VDR expression* in vivo *and found intestinal VDR increased significantly after probiotic colonization compared to the ex-germ-free pig. Furthermore, our unpublished data indicate that probiotics did not inhibit inflammation in mice lacking VDR. 

The presence of VDR in various tissues along with its ability to exert diverse actions in differentiation, growth, and anti-inflammation sets the stage for exploitation of VDR ligands for the treatment of various inflammatory conditions [157, 158]. Although the potential importance of VDR as a therapeutic target has been appreciated [159], no approach to date has safely and effectively altered VDR's activity. Hence, understanding VDR's contribution to probiotic-induced anti-inflammation may provide significant insight in the pathogenesis of inflammatory conditions such as IBD, and thereby, guide the development of novel treatments. Further investigation of the complex interplay of nuclear receptors, defensins, probiotics, and inflammatory pathways may provide significant insight into the mechanisms of action of probiotics in anti-inflammation.

## 7. Current Problems and Future Directions

The individual diversity of the intestinal microflora underscores the difficulty of identifying the entire human microbiota and poses barriers to this field of research. In addition, it is apparent that the actions of probiotics are species and strain specific [19]. It is also apparent that even a single strain of probiotic may exert its actions via multiple, concomitant pathways. Current investigation into the mechanism of action of specific probiotics has focused on probiotic-induced changes in the innate immune functions involving TLRs and its downstream systems including NF-*κ*B, JAK-STAT, MAPK, and SAPK/JNK pathways. Future research on novel mechanisms of action for probiotics involving nuclear receptor signaling, including PPAR*γ* and VDR, is needed. With evolving knowledge, effective probiotic therapy will be possible in the future. 

## Figures and Tables

**Table 1:** Summary for molecular mechanisms and probiotics.

Involved pathways	Probiotics	*In vitro* system	*In vivo* system	Summary	Ref.
NF-*κ*B	(i)* L. casei* Shirota (LcS)	(i) THP-1	(i) Rat	(i) L-lactic acid and LcS culture supernatant inhibited NF-*κ*B activation, TNF-*α* mRNA expression increase, and TNF-*α* protein secretion in cells treated with lipopolysaccharide (LPS)	[33]

NF-*κ*B	(i) LGG	(i) CaCo-2		(i) LGG reduced TNF-*α*-induced NF-*κ*B translocation, lessened decrease in I*κ*B, reduced TNF*α*-induced interleukin (IL)-8 production	[34]

NF-*κ*B	(i) VSL3#	(i) YAMC		(i) VSL#3 inhibited the proinflammatory NF-*κ*B pathway and induced the expression of cytoprotective heat shock proteins (HSPs) in intestinal epithelial cells	[35]

NF-*κ*B	(i)* L. plantarum* (LP)	(i) YAMC, RAW264.7 (ii) Murine dendritic cells		(i) LP-conditioned media (CM) inhibited the chymotrypsin-like activity of the proteasome, NF-*κ*B binding activity as well as the degradation of I*κ*B*α*	[36]

NF-*κ*B	(i) LP-L2	(i) CaCo-2		(i) Caco-2 cells preincubated with LP-L2 showed attenuation of monocyte chemotactic protein-1(MCP-1) protein production and mRNA expression and also prevented I*κ*B*α* degradation in TNF-*α*-stimulated Caco-2 cells	[37]

NF-*κ*B	(i)* L. casei *(Lc)	(i) HEK-293T (ii) CaCo-2		(i) Lc downregulated the transcription of genes encoding proinflammatory effectors and adherence molecules induced by invasive *S. flexneri* resulting in anti-inflammation mediated by the inhibition of the NF-*κ*B pathway	[38]

NF-*κ*B	(i)* B. lactis *	(i) Mode-K (ii) MEF		(i)* B. lactis* activates NF-*κ*B RelA and p38 MAPK phosphorylation in IEC lines	[39]

NF-*κ*B MAPK (ERK1/2/p38/JNK)	(i)* S. boulardii *(Sb)	(i) T84	(i) NIH Mice	(i)* In vivo*, Sb protected mice from *Salmonella enterica* serovar-Typhimurium-(ST-) induced death and prevented hepatic bacterial translocation (ii) In T84 human colorectal cancer cells, Sb incubation abolished *Salmonella* invasion, preserved barrier function, and decreased ST-induced IL-8 synthesis (iii) Sb had an inhibitory effect on ST-induced activation of the MAPKs, extracellular regulated kinase (ERK) 1/2, p38 and JNK, and NF-*κ*B	[40]

NF-*κ*B MAPK (p38/ERK) SAPK/JNK	(i) *E. coli Nissle 1917 *(ECN) (ii)* L. fermentum * (iii)* L. acidophilus *(La) (iv)* Pediococcus pentosaceus * (v)* L. paracasei * (vi) VSL#3	(i) CaCo-2		(i) CaCo-2 cells treated with probiotic had peak of human *β* defensins-2 (hBD-2) mRNA expression at 6 h incubation (ii) Promoter activation via probiotics was abolished with the deletion of NF-*κ*B- and activator-protein-1- (AP-1-) binding sites on the hBD-2 promoter (iii) Induction of hBD-2 depends on MAPK, ERK 1/2, p38, and c-Jun N-terminal kinase (JNK), to varying degrees	[41]

NF-*κ*B SAPK/JNK	(i) ECN (ii) Various *E. coli strains * (iii) Various *Lactobacilli strains *	(i) CaCo-2		(i) Active and heat-inactivated ECN and several other probiotic bacteria potently induced hBD-2 in intestinal epithelial cells (ii) hBD-2 promoter activation was abolished by mutation of the two NF-kB sites in the hBD-2 promoter upon treatment with ECN (iii) ECN-inducted activation of AP-1 may be regulated by JNK kinase pathway	[42]

SAPK/JNK MAPK (p38)	(i) LGG	(i) YAMC		(i) LGG-CM induced Hsp25 and Hsp72 in time- and concentration-dependent manner (ii) LGG-CM-induced HSP72 induction was blocked by the Inhibitors of p38 and JNK	[43]

Epidermal growth factor receptor (EGFR)	(i) Sb	(i) HT-29	(i) APC min	(i) Upon exposure to Sb, HER-2, HER-3, insulin-like growth factor-1 receptor, and EGFR were inactivated (ii) In HT-29 cells, Sb promoted apoptosis, prevented EGF-induced proliferation, and reduced cell colony formation (iii) Sb decreased intestinal tumor growth and dysplasia in C57BL/6J Min/+ (Apc(Min)) mice	[44]

TLR NF-*κ*B MAPK (p38)	(i) LGG (ii)* B. longum *	(i) HT-29 (ii) T84		(i) Commensal-origin DNA enhanced expression of TLR9 in HT-29 and T84 cells (ii) This was associated with attenuation of TNF-*α*-induced NF-*κ*B activation by reducing I*κ*B*α* degradation and p38 phosphorylation (iii) LGG DNA decreased the TNF-*α*-induced reduction in transepithelial electrical resistance (TER)	[45]

TLR	(i) *LP BFE 1685 * (ii) LGG	(i) HT-29		(i) HT29 cells incubated with lactobacilli showed upregulation of TLR2 and TLR9 transcription levels (ii) Protein expression levels of TLR2 and TLR5 were enhanced	[46]

TLR	(i) VSL#3		(i) Mice	(i) Intragastric administration of gamma-irradiated probiotics significantly ameliorated the severity of DSS-induced colitis in TLR2- and TLR4-deficient mice but not in TLR9-deficient mice	[47]

TLR	(i) ECN		(i) Wild-type (WT), TLR2-/TLR4-knockout mice	(i) ECN decreased colitis and proinflammatory cytokine secretion in WT but not TLR2- or TLR4-knockout mice (ii) ECN resulted in reduction of interferon (IFN)-*γ* secretion in TLR2 knockout (iii) Cytokine secretion was almost undetectable and not modulated by ECN in TLR-4-knockout mice (iv) Increased TLR2 and TLR4 protein expression and NF-*κ*B activity via TLR2 and TLR4 was seen with ECN and human T-cell coculture	[48]

MAPK (p38/ERK)	(i) LP (ii) Lc	(i) Mice peritoneal macrophages		(i) LP strongly induced IL-10 and weakly induced IL-12; Lc strongly induced IL-12 and weakly induced IL-10 (ii) LP, compared to Lc, demonstrated more rapid and strong activation of MAPKs, especially of ERK (iii) Blocking LP-induced ERK activation resulted in decreased IL-10 production and increased IL-12 production (iv) Combined stimulation with LP and Lc resulted in synergistic induction of IL-10 production; this was triggered by the key factors: cell wall teichoic acid and lipoteichoic acids (v) Teichoic-acid-induced IL-10 production was mediated by TLR2-dependent ERK activation	[49]

MAPK (p38/ERK) Tight junctions (TJ)	(i) LGG	(i) CaCo-2		(i) LGG-produced soluble proteins (p40 and p75) diminished the hydrogen-peroxide-induced decrease in TER and increase in inulin permeability and induced increase in membrane translocation of protein kinase C (PKC) beta I, PKC epsilon, and level of phospho-ERK1/2 in the detergent-insoluble fractions (ii) LGG-produced soluble proteins (p40 and p75) prevented hydrogen-peroxide-induced redistribution of occludin, zonula occludens (ZO)-1, E-cadherin, and beta-catenin from intercellular junctions and their dissociation from the detergent-insoluble fractions (iii) p40- and p75-mediated reduction of hydrogen-peroxide-induced tight junction disruption and inulin permeability was attenuated by U0126 (a MAP kinase inhibitor)	[50]

MAPK (p38/ERK) TJ	(i)* B. infantis *	(i) T84	(i) IL-10, IL-1 deficient mice	(i) *B. infantis*-conditioned medium (BiCM) increased TER, ZO-1, and occludin expression and decreased claudin-2 expression; this was associated with increased phospho-ERK and decreased phospho-p38 (ii) TNF-*α*- and IFN-*γ*-induced drops in TER and rearrangement of TJ proteins were prevented by BiCM (iii) Inhibition of ERK attenuated the protection from TNF-*α* and IFN-*γ* and prevented BiCM-induced increase in TER (iv) *In vivo*, oral BiCM reduced colonic permeability, in IL-10-deficient mice, long-term BiCM decreased colonic and splenic IFN-*γ* secretion, attenuated inflammation, and normalized colonic permeability	[51]

TJ	(i) LP MB452	(i) CaCo-2		(i) LP MB452 increased TER across Caco-2 cell monolayers in dose-dependent manner (ii) Altered expression of several tight-junction-related genes (including occludin and associated plaque proteins) was seen in response to LP MB452 (iii) LP MB452 caused changes in gene expression levels of tubulin and proteasome (iv) LP MB452-treated cells showed increased fluorescence intensity of the four tight junction proteins when compared to untreated controls	[52]

TJ PKC	(i) LP	(i) CaCo-2		(i) Unconjugated bilirubin (UCB) caused decreased PKC activity, serine phosphorylated occluding, and ZO-1 levels (ii) High concentrations of UCB caused cytotoxicity and decreased TER (iii) Treatment with LP mitigated the effects of UCB on TER and apoptosis, prevented aberrant expression and rearrangement of TJ proteins, and partially restored PKC activity and serine phosphorylated TJ protein levels	[53]

TJ	(i) LP DSM2648	(i) CaCo-2		(i) LP DSM 2648 reduced the deleterious effect of *Escherichia coli* (enteropathogenic *E. coli* (EPEC)) O127:H6 (E2348/69) on TER and adherence with simultaneous or prior coculture compared with EPEC incubation alone	[54]

TJ TLR	(i) LP	(i) CaCo-2	(i) Human	(i) LP induced translocation of ZO-1 to the TJ region in an *in vitro* model, but the effects on occludin were minor compared with effects seen *in vivo * (ii) LP activated TLR2 signaling, and treatment with TLR2 agonist Pam(3)-Cys-SK4(PCSK), increased fluorescent staining of occludin in the TJ (iii) Phorbol-ester-induced dislocation of ZO-1 and occludin and associated increase in epithelial permeability were attenuated with pretreatment with LP or PCSK	[55]

TJ	(i) *B. bifidum *		(i) Rats	(i) *B. bifidum* decreased the incidence of necrotizing enterocolitis (NEC), normalized IL-6, mucin-3, and Tff3 levels in the ileum of NEC rats, and normalized the expression and localization of TJ and adherens junction (AJ) proteins in the ileum compared with animals with NEC (ii) *B. bifidum* did not affect reduced mucin-2 production in the NEC rats	[56]

TJ	(i) VSL#3		(i) BALB/c mice	(i) VSL#3 treatment prevented the increase in epithelial permeability in acute colitis, decrease in expression and redistribution of occludin, ZO-1, and claudin-1, claudin-3, claudin-4, and claudin-5, and increase of epithelial apoptotic ratio	[57]

TJ	(i) Lr (ii) La		(i) Mice	(i) Pretreatment with combination of Lr and La significantly prevented decrease in the membrane-bound ATPases and reduced expression of tight junction proteins in the membrane	[58]

TJ	(i) *B. lactis 420 * (ii) *B. lactis HN019 * (iii) La* NCFM * (iv) *L. salivarius Ls-33 *	(i) CaCo-2		(i) *B. lactis* 420 and *Escherichia coli* O157:H7 (EHEC) supernatant had opposite effects in tight junction integrity; * B. lactis* 420 supernatant protected the tight junctions from EHEC-induced damage when administered before EHEC supernatant (ii) EHEC and probiotics had reverse effects upon cyclo-oxygenase expression	[59]

TJ	(i) ECN		(i) BALB/c mice	(i) ECN colonization of gnotobiotic mice resulted in upregulation of ZO-1 mRNA and protein levels in IECs (ii) ECN administration reduced loss of body weight and colon shortening in DSS-treated mice (iii) ECN inoculation ameliorates the infiltration of the colon with leukocytes	[60]

TJ PKC	(i) ECN	(i) T84		(i) EPEC with ECN coincubation: addition of ECN after EPEC infection restored barrier integrity and abolished barrier disruption (ii) ECN altered the expression, distribution of ZO-2 protein and of distinct PKC isotypes; ZO-2 expression was increased in parallel to its redistribution towards the cell boundaries (iii) ECN induces restoration of a disrupted epithelial barrier; this is transmitted by PKCzeta silencing and ZO-2 redistribution	[61]

TJ	(i) LGG	(i) MDCK-1, T84		(i) EHEC-induced decrease in electrical resistance and the increase in barrier permeability assays were attenuated by probiotic pretreatment (ii) LGG protected epithelial monolayers against EHEC-induced redistribution of claudin-1 and ZO-1 proteins (iii) Heat-inactivated LGG did not affect EHEC binding or disruption of barrier function	[62]

JAK/STAT MAPK (p38/ERK)	(i)* Streptococcus thermophilus * (ii) La	(i) HT29/cl.19A (ii) Caco-2,		(i) *Streptococcus thermophilus* (ST)/La or the commensal *Bacteroides thetaiotaomicron* (BT) prevented the TNF-*α*- and IFN-*γ*-induced reduction in TER and increase in epithelial permeability (ii) ST/La or BT prevented IFN-*γ* inhibition of agonist-stimulated chloride secretion (iii) ST/La or BT restoration of Cl(-) secretion was blocked by inhibitors of p38 MAPK, ERK1, 2, and PI3K (iv) ST/La pretreatment reversed the IFN-*γ*-induced downregulation of the cystic fibrosis transmembrane conductance regulator (CFTR) and the NKCC1 cotransporter (v) The effects of ST/La or BT on TER and permeability were potentiated by a Janus kinase (JAK) inhibitor but not by p38, ERK1, 2, or PI3K inhibition (vi) Reduced activation of suppressor of cytokine signaling (SOCS)3 and STAT1,3 was seen only in probiotic-treated epithelial cells exposed to cytokines	[63]

JAK/STAT NF-*κ*B	(i) LcS	(i) RAW264.7 (ii) LI-LPMC (iii) UC-PBMC	(i) SAMP1/Yit mice	(i) LcS improved chronic ileitis in SAMP1/Yit mice (ii) Lcs improved murine chronic colitis, and this was associated with decreased IL-6 production by large intestinal lamina propria mononuclear cells ( LI-LPMCs) and downregulation of proinflammatory cytokines such as IL-6 and IFN-*γ* production in LPMC (iii) IL-6 release in LPS-activated LI-LPMC, RAW264.7, and ulcerative colitis-peripheral blood mononuclear cells (UC-PBMCs) was inhibited by LcS-derived polysaccharide-peptidoglycan complex (PSPG) (iv) In lipopolysaccharide- (LPS-) stimulated LI-LPMC and RAW264.7 cells, LcS inhibited the production of IL-6; other strains of *Lactobacillus* did not (v) Nuclear translocation of NF-*κ*B was downregulated by LcS	[64]

JAK/STAT	(i) *Lactobacillus helveticus *R0052	(i) Intestine 407 (ii) HEp-2 (iii) Caco-2		(i) After EHEC O157:H7 infection, STAT-1 activation was reduced compared to uninfected cells (ii) Preincubation with *L. helveticus* R0052 (but not boiled *L. helveticus* R0052, an equal concentration of viable Lr R0011, or surface-layer proteins) followed by EHEC infection abrogated disruption of IFN-*γ*-STAT-1 signaling	[65]

SAPK/JNK MAPK (p38) NF-*κ*B	(i) *S. cerevisiae *UFMG 905		(i) Mice	(i) After *Salmonella* challenge, *S. cerevisiae* 905 inhibited weight loss and increased survival rate (ii) Levels of proinflammatory cytokines were decreased, and activation of mitogen-activated protein kinases (p38 and JNK, but not ERK1/2), NF-*κ*B, and AP-1 was modulated by *S. cerevisiae* 905	[66]

Lipid/Xenobiotic	(i) VSL#3		(i) IL-10 KO	(i) Probiotics resulted in downregulation of CXCL9, CXCL10, CCL5, T-cell activation and IRGM (ii) Probiotic treatment decreased the number of CCL5+ CD3+ double-positive T cells consistent with reduction in integrins and upregulated galectin2 (iii) Lipid- and PPAR-signaling-associated genes were also upregulated (iv) Altered microbial diversity was noted in probiotic-treated mice (v) Inflammation in IL10-KO mice showed differential regulation of various signaling pathways( inflammatory, nuclear receptor, lipid, and xenobiotic)	[67]

Metabolism/ Glucose Uptake	(i) La (ii) *L. gasseri * (iii) *L. amylovorus * (iv) *L. gallinarum * (v) *L. johnsonii *	(i) CaCo-2		(i) Exposure to bacteria-free supernatants of La cultured in chemically defined media (CDM) with 110 mM fructose increased glucose accumulation; exposure to a suspension of the bacteria had no effect (ii) Heat-denaturing the supernatant diminished the increase in glucose accumulation (iii) Supernatants prepared with anaerobic culture of *L. gasseri, L. amylovorus, L. gallinarum, and L. johnsonii *in the CDM with fructose increased glucose accumulation	[68]

Reactive Oxygen Species (ROS) NF-*κ*B	(i) Lr	(i) FHs74Int	(i) C57BL/6J	(i) LGG induced ROS generation in intestinal epithelia *in vitro* and *in vivo * (ii) Increased glutathione (GSH) oxidation and cullin-1 deneddylation was seen in the intestines of mice fed LGG, showing local ROS generation and the resultant Ubc12 inactivation (iii) Prefeeding LGG prevented TNF-*α*-induced activation of intestinal NF-*κ*B	[69]

## Abbreviations

AAD: Antibiotic-associated diarrhea
AP-1: Activator protein-1
CD: Crohn's disease
CDAD: *Clostridium-difficile*-associated disease
CDM: Chemically defined media
CM: Conditioned media
CTFR: Cystic fibrosis transmembrane conductance regulator
ECN: *E. coli* Nissle 1917
EGFR: Epidermal growth factor receptor
EHEC: *E. coli* O157:H7
EPEC: Enteropathogenic *E. coli *
ERK: Extracellular-signal-regulated kinase
GSH: Glutathione
hBD: Human beta defensin
HSP: Heat shock proteins
IBD: Inflammatory bowel disease
IBS: Irritable bowel syndrome
IEC: Intestinal epithelial cell
IFN: Interferon
IL: Interleukin
I*κ*B: Inhibitor of kappa B
JAK/STAT: Janus kinase/signal transducers and activators of transcription
La: *Lactobacillus acidophilus*
Lc: *Lactobacillus casei*
LcS: *Lactobacillus casei* Shirota
LGG: *Lactobacillus rhamnosus* GG
LI-LPMC: Large intestinal lamina propria mononuclear cells
LP: *Lactobacillus plantarum*
LPS: Lipopolysaccharide
Lr: *Lactobacillus rhamnosus*
MAPK: Mitogen-activated protein kinase
MCP: Monocyte chemotactic protein
NEC: Necrotizing enterocolitis
NF-*κ*B: Nuclear factor kappa B
PKC: Protein kinase C
PPAR*γ*: Peroxisome-proliferator-activated receptor gamma
ROS: Reactive oxygen species
RXR: Retinoid X receptor
SAPK/JNK: Stress-activated protein kinase/c-Jun NH2-terminal kinase
Sb: *Saccharomyces boulardii*
SOCS: Suppressor of cytokine signaling
TCR: T-cell receptor
TER: Transepithelial electrical resistance
TJs: Tight junctions
TLR: Toll-like receptor
TNF: Tumor necrosis factor
UC: Ulcerative colitis
UCB: Unconjugated bilirubin
VDR: Vitamin D receptor
ZO: Zonula occludens.
